# Comparative effectiveness of different modes of exercise interventions in diabetics with frailty in China: a systematic review and a network meta-analysis

**DOI:** 10.1186/s13098-023-01248-x

**Published:** 2024-02-26

**Authors:** Liu Qin, Peng Junjie, Wei Xinhong, Fang Shengju, Sun Ruifen

**Affiliations:** 1 School of Government, Yunnan University, Kunming, 650504 China; 2 School of Nursing, Yunnan University of Chinese Medicine, Kunming, 650500 China

**Keywords:** Diabetes, Frailty, Intervention, Systematic review, Randomized controlled trial, Network meta-analysis, China

## Abstract

**Objective:**

To systematically evaluate the efficacy of different training modes in patients with diabetes decline.

**Methods:**

PubMed, Cochrane Library, EMbase, Web of Science, CNKI, VIP, WANFANG, SinoMed were searched in computer to collect randomized controlled trials (RCTs) of training intervention in patients with diabetes and frailty, and the search time was as of May 21, 2023. After two review authors independently screened studies, extracted data, and assessed the risk of bias of included studies, network meta-analysis was performed using Stata14.0 and R4.3.1 software. Fasting blood glucose (FGB), glycosylated haemoglobin (HbA1c), two-hour postprandial blood glucose (PBG), total cholesterol (TCH), triglycerides (TG), low-density lipoprotein cholesterol (LDL-C), Short Physical Performance Battery (SPPB), and body mass index (BMI) were used as outcome measures.

**Results:**

A total of 15 RCTs were included, including 1550 patients. The results of the network meta-analysis showed that integrated training reduced FBG compared with the control group; integrated training, Pilates training, resistance training can reduce HbA1c; Pilates training and resistance training can reduce PBG; integrated training, Pilates training, resistance training can reduce TCH; Pilates training and resistance training can reduce TG; resistance training improves BMI. The results of the best probability ranking showed that multi-group training had the most significant effect on improving PBG and SPPB scores.

**Conclusion:**

The current evidence suggests that multi-group training is the best way to reduce fasting blood glucose and improve physical activity before meals, and Pilates training may be the best way to reduce glycated hemoglobin, blood glucose two hours after meals, improve blood lipid level and BMI in patients with diabetes in China.

*Trial registration*: PROSPERO registration number for this study: CRD42023427868.

**Supplementary Information:**

The online version contains supplementary material available at 10.1186/s13098-023-01248-x.

## Introduction

Diabetes is a worldwide health problem. The latest data from the International Diabetes Federation (IDF) in 2021 showed that the number of people with diabetes in the world was 537 million, which is expected to increase to 643 million by 2030 [1]. In China, the prevalence of diabetes in the adult population increased from 10.4% in 2013 to 11.2% in 2015 – 2017 [2], and the number of patients ranks first in the world. In 2021, there were more than 140 million diabetics in China, and 30% of them were elderly [3]. The combination of factors such as aging, urbanization, and the increasing prevalence of overweight and obesity will further increase the pressure on diabetic patients in China, which will put tremendous pressure on Chinese healthcare system. The health problems of elderly diabetic patients will become more prominent in the future.

Older people with diabetes are prone to frailty. Frailty is a clinical syndrome independent of normal aging, the core of which is the cumulative decline of multiple physiological systems, resulting in insufficient physiological reserves and reduced ability to resist adverse stimuli, making a lesser number of stimuli sufficient to cause adverse events [4]. The health of the elderly declines with age, but there are certain differences in the health status of different elderly patients of the same age, and frailty is thought to be the cause of this difference [5]. At the same time, the importance of frailty is also recognized by guidelines on diabetes management [6].

Compared with people without diabetes, people with diabetes have a higher risk of frailty. Some studies [7] have pointed out that the incidence of frailty in elderly diabetic patients is about 3–5 times higher than that in non-diabetic patients. A longitudinal study conducted by Chhetri et al. [8] showed that the prevalence of frailty in diabetic patients (19.32%) was much higher than that in pre-diabetic patients (11.92%) and non-diabetic patients (11.43%), and the prevalence of frailty increased with age, reaching 42.31% over the age of 85. Although the mechanism between frailty and diabetes is not fully understood, researchers believe that inflammatory states, oxidative stress, mitochondrial dysfunction, malnutrition, and different energy imbalances are the bridges between the two [9, 10], with sarcopenia playing an important role in this process. Diabetes-related hormone deficiency [11], neuropathy [12], chronic inflammation [13] and insulin resistance [14] can slow down protein synthesis and increase muscle loss, which will increase the incidence of sarcopenia in patients, and accelerate the occurrence of frailty [7]. In addition, patients with diabetes are often at risk of malnutrition, which will lead to the decline of muscle and bone quality, weaken the body’s immunity, reduce the body’s activity, and make patients prone to frailty symptoms [15].

Patients with diabetes and frailty are more likely to have adverse health outcomes such as fractures, falls, disability, readmission to hospital and reduced daily activities, and even death [5, 16–19]. At the same time, frailty may increase the risk of complications and hypoglycemia [19, 20], which can be dangerous for the long-term health of people with diabetes. For the patient's family, frailty can increase financial stress and the burden of home care. Therefore, the health problems of patients with diabetes mellitus and frailty need to be paid attention to.

Frailty is considered reversible by the investigators, which makes it important for early intervention in frail patients [21]. Among the various interventions, exercise interventions are one way that is valued. Exercise is an important cornerstone in the treatment of frailty, and people with diabetes have the need to control their blood sugar through exercise. Therefore, for diabetic patients with frailty at the same time, exercise is an important measure to improve their health. It can help to control blood glucose fluctuations by increasing insulin sensitivity, improve the level of body function, enhance muscle mass, improve flexibility and balance, which is conducive to the improvement and treatment of frailty. Studies have shown that regular training is a protective factor for diabetic patients with frailty [8], Morley et al. [22] found that diabetic patients who adhered to aerobic program for a long time were more likely to improve their frailty. At present, some studies have proved that exercise interventions are effective for patients with frailty and patients with diabetes [23–25]. Various medical organizations have provided their advice on the choice of exercise for diabetic patients [26–29]. When it comes to exercise recommendations for frail patients, there seems to be no universal answer to the recommended best form of exercise [30, 31].

Therefore, current studies have not yet indicated which exercise provides the best training method for the crossover of patients with diabetes mellitus and frailty. Network Meta-analysis can evaluate the effect of each intervention by comparing the effect of research. Therefore, this study uses the method of network meta-analysis to evaluate the effect of each training intervention and summarize the best training mode.

## Methods

### Search strategy

PubMed, Cochrane Library, Embase, Web of Science, CNKI, WANFANG, VIP and SinoMed were searched by computer. Randomized controlled trials (RCTs) in patients with both diabetes and frailty were collected. The search time limit was from the establishment of the database to May 21, 2023, and the language type was Chinese or English. In addition, the references of the included literature were traced back to supplement the acquisition of relevant literature. The search was conducted by combining subject words and free words. (Additional file 1: Annexes 1).

### Inclusion and exclusion criteria

#### Study Type:

Randomized controlled trials (RCTs) involving patients with both diabetes and frailty were considered eligible

#### Subjects:

Individuals meeting the following criteria were included

(1) Diagnosed with diabetes;

(2) Diagnosed with frailty;

(3) Research conducted in China.

#### Interventions:

All combined or individual exercise interventions were eligible for inclusion

#### Outcome Measures

(1) Blood Glucose: Fasting blood glucose (FBG); glycosylated haemoglobin (HbA1c), 2-h postprandial blood glucose(PBG);

(2) Blood Lipids: Total cholesterol (TCH), triglycerides (TG), low-density lipoprotein cholesterol (LDL-C);

(3) Physical Activity: Short Physical Performance Battery (SPPB);

(4) Physical Status: Body Mass Index (BMI).

#### Exclusion criteria

(1) The language of the article is not in English or Chinese;

(2) Studies for which data could not be extracted for analysis;

(3) Duplicate published studies;

(4) Review, conference, letter type articles.

### Data extraction

Study selection and data extraction records identified from databases and manual searches were imported into Endnote X9.0. According to the inclusion and exclusion criteria, two investigators respectively evaluated and screened the titles and abstracts recorded in Endnote X9.0 to finally determine the included literature. Analyses were resolved by discussion between the two authors, and a third author was invited to adjudicate when necessary. Data extraction included: (1) RCT characteristics (e.g., authors, year of publication, study location, sample size, intervention and control measures); (2) characteristics of the participants (e.g., gender, age); And (3) outcomes (e.g., FBG, HbA1c, PBG). For multiple articles with data from the same RCT, the article with the longest follow-up period was selected, and the remaining articles were supplemented. For multiple sets of experiments, each set of data will be extracted.

### The risk of bias of the included studies

The Cochrane risk of bias tool for randomised trials (RoB 2) was used to assess the inexpensive risk of RCTs [32]. Two researchers independently evaluate the quality of the article, and when there is a disagreement between the two researchers, a third researcher participates in the discussion and helps to resolve the disagreement.

### Statistical analysis

The network package of Stata 14.0 and the netmeta package of version 2.8–2 of R4.3.1 were used in this study. If the outcome was a continuous variable, the mean difference (MD) was used as the effect size indicator and the 95% confidence interval (*CI*) was provided. In R4.3.1 software, the netmeta package of 2.8–2 is used for data analysis. The level of heterogeneity between studies was assessed using the *I*^2^ statistic, and when *I*^2^ > 50%, the heterogeneity between studies was considered statistically significant, in which case a random-effects model was used; otherwise, a fixed-effect model was used [33]. In the stata 14.0 software, the network package is used for data analysis. Heterogeneity between studies was assessed by calculating Cochran’s Q statistic, and a fixed-effect model was chosen when the P value of the Q statistic was > 0.05; otherwise a random-effects model was used [34].

For data expressed in quartiles and medians, conversion to the format of mean and standard deviation [35, 36].When P < 0.05, the difference was considered statistically significant. Node analysis was used for inconsistency test, and if P > 0.05, the consistency model was used for analysis. Otherwise, the inconsistency model was used. The effect of exercise intervention was ranked by the best probability ranking chart.

### Sensitivity analysis and consistency tests

Pooled data using fixed-effect and random-effects models were used to assess the robustness of network meta-analysis. We use the global inconsistency method for inconsistency testing, and the node-splitting method for local inconsistency. If the P > 0.05, there is no significant difference in the results, and the consistency model is used, otherwise, the inconsistency model is used.

### Publication bias

Funnel plots were used to test the included literature for publication bias for each outcome [37].

## Results

### Literature selection

**Fig. 1:**
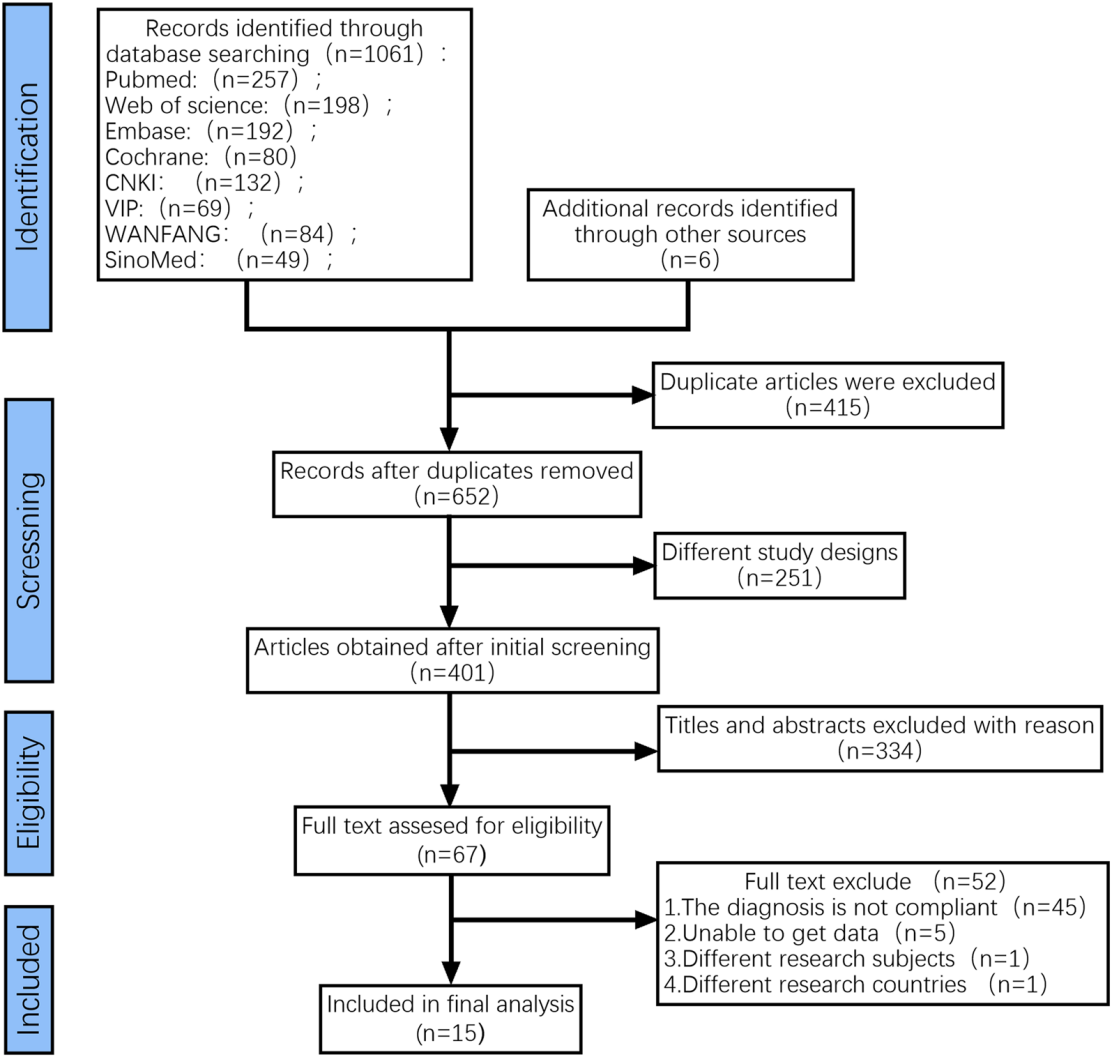
Document screening process and results

The search revealed a total of 1067 studies in various databases. These included PubMed (257), Cochrane Library (198), Embase (192), Web of Science (80), CNKI (132), VIP (69), WANFANG (84), SinoMed (49), and 6 articles retrieved by other sources. After screening, 415 duplicate studies were excluded, 251 studies were excluded because their content did not match this study, and 334 studies were excluded after reading the title and abstract sections. After reading the remaining 67 studies in full, 52 studies were excluded (45 studies lacked a basis for the diagnosis of frailty or diabetes, 5 studies were unable to extract data, 1 study did not meet study population requirements and 1 study did not meet regional requirements). As a result, a total of 15 RCTs were included in this study. (Fig. 1).

### Characteristics of the included studies

The basic characteristics of the included studies are shown in Table 1. A total of 1550 patients were included in our network meta-analysis. All studies included in this study included two arms. The included studies were published between 2019 and 2023 and had an intervention duration of 2 to 6 months. There are five main types of training in the literature: resistance training, aerobic training, comprehensive training, Otago training, and Pilates training. (Table 1).

**Table 1:** Characteristics of included studies

Author, year	Region	Sample size	Age	Male and female	Duration	Training modality	Comparator	Outcome measures
Zhou [38]	Guangxi	70	≥ 60	55/15	3 months	Integrated training	Usual treatment	①②③
Cui [39]	Henan	90	≥ 60	55/35	3 months	Pilates training	Usual treatment	①②③
Zhang [40]	Henan	89	≥ 60	26/63	3 months	Multi-group training	Usual treatment	⑦
Gu [41]	Xingjiang	106	≥ 65	62/44	6 months	Otago training	Usual treatment	⑦
Hu [42]	Beijing	95	≥ 65	46/39	3 months	Integrated training	Usual treatment	①②③
Fang [43]	Zhejiang	80	67.9 ± 4.3/68.2 ± 4.8	22/58	3 months	Integrated training	Usual treatment	①④⑤⑥⑦
Qian [44]	Gunagdong	168	≥ 60	102/66	2 months	Integrated training	Usual treatment	①②④⑤⑥⑧
Liu [45]	Fujian	110	65 ~ 75	58/52	6 months	Integrated training	Usual treatment	①②③
Liu [46]	Hunan	86	≥ 60	41/45	12 weeks	Otago training	Usual treatment	①
Zhang [47]	Jiangsu	56	≥ 60	39/17	2 months	Pilates training	Usual treatment	①②③④⑤⑥⑧
Chen [48]	Hebei	191	≥ 60	117/74	3 months	Resistance training	Usual treatment	①②③④⑤⑥⑧
Wang [49]	Fujian	80	55.14 ± 3.16/54.85 ± 3.21	46/34	3 months	Integrated training	Usual treatment	①②
Jin [50]	Jiangsu	172	≥ 60	99/73	3 months	Resistance training	Usual treatment	①②③④⑤⑥⑧
Zeng [51]	Gunagdong	68	54.69 ± 1.54/55.27 ± 10.88	41/28	3 months	Resistance training	Usual treatment	①②③⑧
Yan [52]	Jiangsu	89	≥ 60	/	12 weeks	Integrated training	Usual treatment	①

①Fasting blood sugar; ② Glycated hemoglobin; ③ Blood glucose two hours after meals; ④ Total cholesterol; ⑤ Triglycerides; ⑥ LDL cholesterol; ⑦ The Short Physical Performance Battery (SPPB); ⑧Body Mass Index (BMI)

### Quality evaluation

**Fig. 2:**
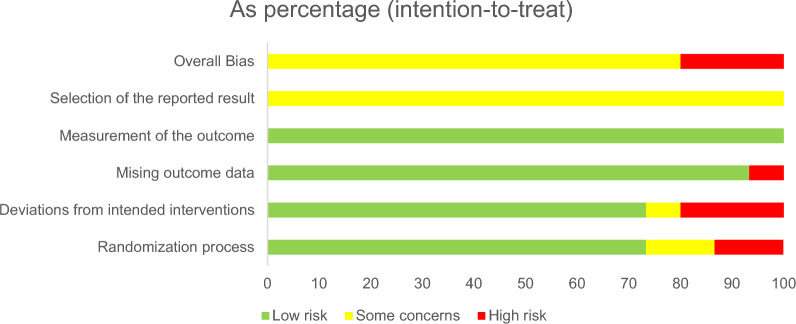
Summary results on risk of bias (using RoB2) of including RCTs

A total of 3 articles were judged to be high risk and 12 articles were judged to be some concerns in this assessment. Of the randomization process evaluations, 2 were judged to be high risk. 3 of the Deviations from intended interventions were rated as high risk. In the evaluation of Mising outcome data, 1 was rated as high risk, and the rest were low risk. All studies were assessed as low risk in the Measurement of the outcome. In the Selection of the reported result, all studies were assessed as having some concerns. (Fig. 2, Additional file 1: Fig. 1: Results on risk of bias (using RoB2) of including RCTs).

### Results of network meta-analysis

#### Fasting blood glucose

**Fig. 3:**
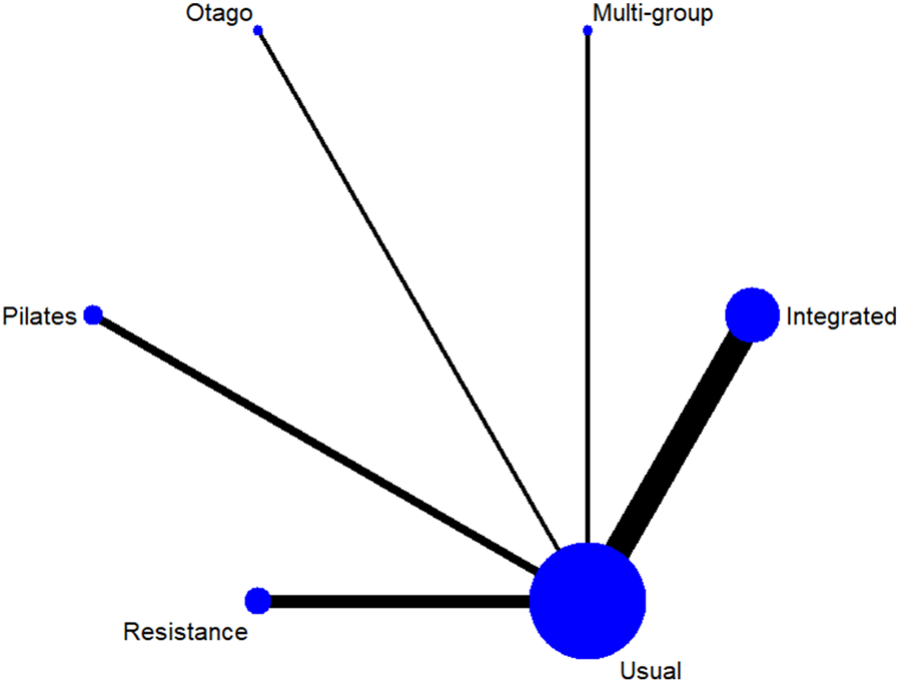
Network diagram of different training on fasting blood glucose in diabetic patients with frailty

A total of 13 studies [38–40, 42–46, 48–52] on fasting blood glucose were included. The results of network meta-analysis showed that the fasting blood glucose of the integrated training group [MD = − 1.13, 95%CI (− 1.80, − 0.51), P < 0.05] was better than that of the control group. However, there was no significant difference in training therapy between any two groups (P > 0.05). The results of the best probability ranking showed that multi-group training had the best effect on reducing fasting blood glucose in diabetic patients with frailty. This suggests that there is a statistically significant difference between integrated training and usual care in improving fasting blood glucose, while multigroup training may have the best efficacy. (Figs. 3 and Table 2 below, Additional file 1: Annexes 1).

**Table 2:**
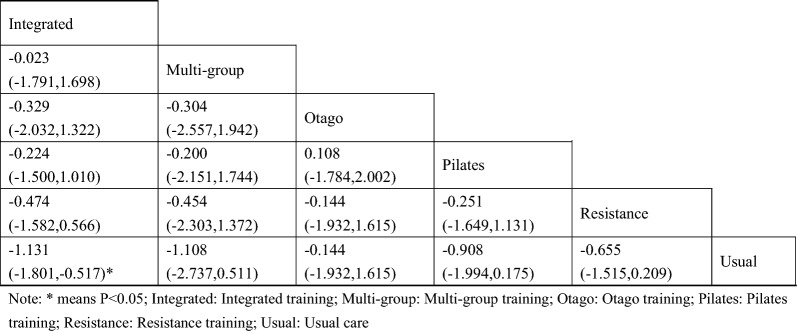
League table of different trainings on fasting blood glucose in diabetic patients with frailty

#### Glycosylated hemoglobin

**Fig. 4:**
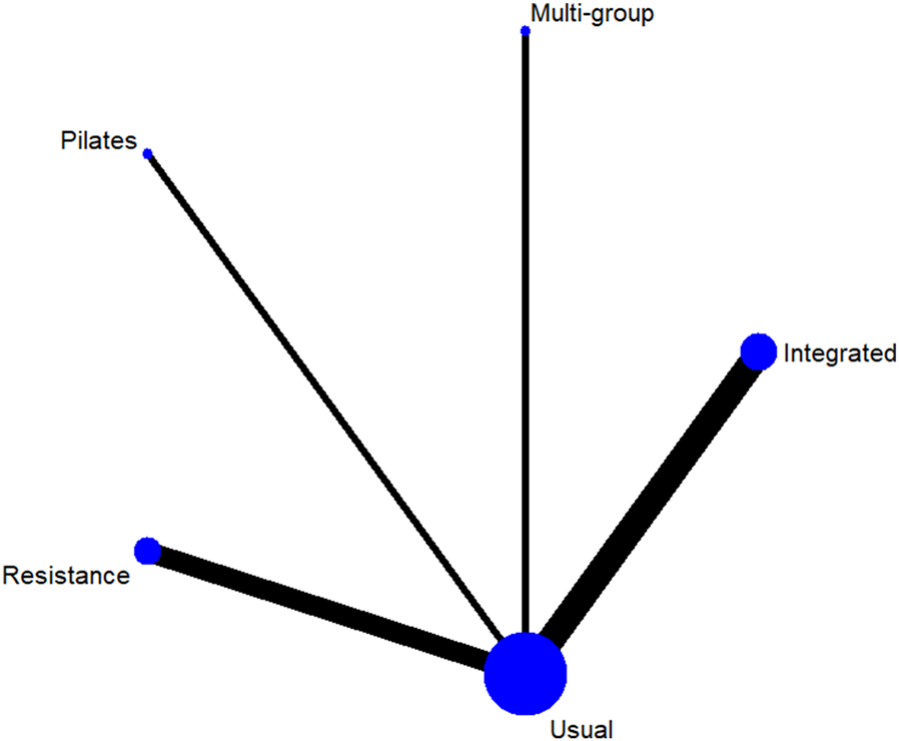
Network diagram of different training on glycated hemoglobin in diabetic patients with frailty

A total of 9 studies on Hba1c were included [38, 39, 42, 44, 45, 48–51]. The results of network Meta-analysis showed that integrated training [MD = − 1.13, 95%CI (− 1.80, − 0.51), P < 0.05], Pilates training [MD = − 1.85, 95%CI (− 2.49, − 1.21), P < 0.05], resistance training [MD = − 0.82, P < 0.05], 95%CI (− 1.18, − 0.45), P < 0.05]. The effect of integrated training [MD = 0.98, 95%CI (0.24, − 1.68), P < 0.05] was worse than that of Pilates training, and the effect of Pilates training on glycated hemoglobin was better than that of resistance training [MD = − 1.02, 95%CI (− 1.77, − 0.29), P < 0.05]. The results of the best probability ranking showed that Pilates training had the best effect on reducing glycosylated hemoglobin in diabetic patients with frailty. This suggests that there are statistically significant differences in glycosylated hemoglobin improvement between integrated training and usual care, between Pilates training and usual care, and between resistance training and usual care, while Pilates training may have the best effect. (Figs. 4 and Table 3, Additional file 1: Annexes 1).

**Table 3:**
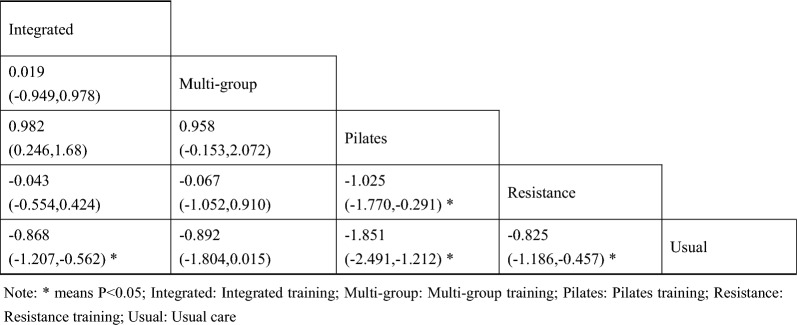
League table of different trainings on glycated hemoglobin in diabetic patients with frailty

#### Two-hour postprandial blood glucose

**Fig. 5:**
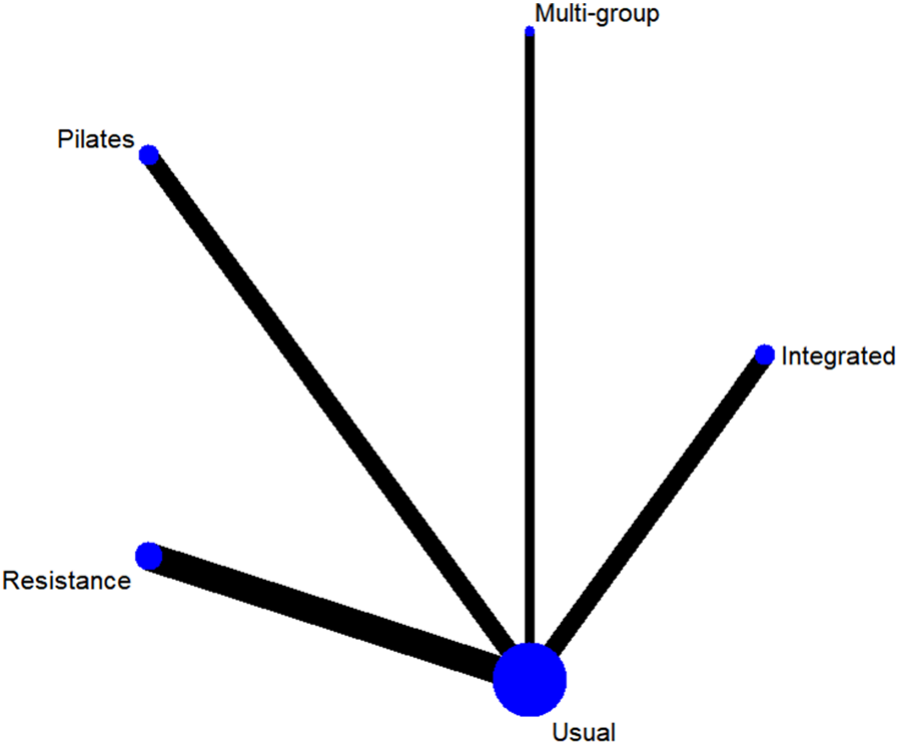
Network diagram of different training on blood glucose two hours after meal in diabetic patients with frailty

A total of 8 studies were included on two-hour postprandial glucose [38, 39, 42, 45, 47, 48, 50, 51]. The results of network meta-analysis showed that the 2-h postprandial blood glucose of Pilates training [MD = − 1.28, 95%CI (− 1.99, − 0.56), P < 0.05] and resistance training [MD = − 0.97, 95%CI (− 1.53, − 0.42), P < 0.05] was better than that of the control group. However, there was no significant difference in training therapy between any two groups (P > 0.05). The results of the best probability ranking showed that Pilates training was the most effective in reducing 2-h postprandial blood glucose in patients with diabetic frailty. This suggests that there are statistically significant differences in postprandial blood glucose improvement between the Pilates training and usual care, and between the resistance training and usual care, with the Pilates training likely having the best efficacy. (Figs. 5 and Table 4, Additional file 1: Annexes 1).

**Table 4:**
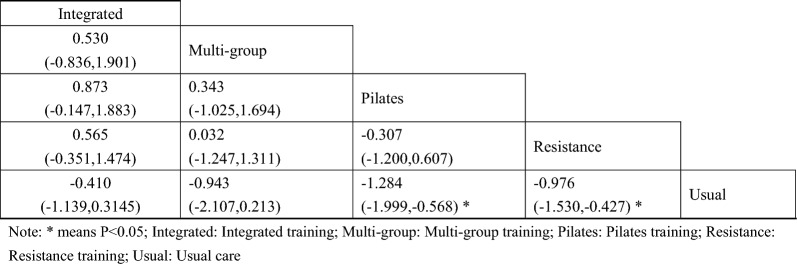
League table of different trainings on blood glucose two hours after meal in diabetic patients with frailty

#### Total cholesterol

**Fig. 6:**
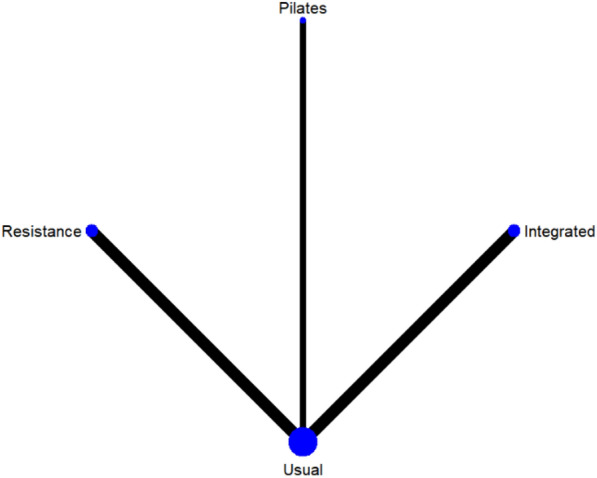
Network diagram of different training on total cholesterol in diabetic patients with frailty

A total of 5 studies on total cholesterol were included [43, 44, 47, 48, 50]. The results of network Meta-analysis showed that integrated training [MD = − 0.70, 95%CI (− 1.29, − 0.24), P < 0.05], Pilates training [MD = − 1.10, 95%CI (− 1.87, − 0.32), P < 0.05], resistance training [MD = − 1.06, P < 0.05], 95%CI (− 1.59, − 0.53), P < 0.05]. However, there was no significant difference in training therapy between any two groups (P > 0.05). The results of the best probability ranking showed that Pilates training had the best effect on reducing total cholesterol in diabetic patients with frailty. This suggests that there were statistically significant differences in total cholesterol improvement between integrated training and usual care, between Pilates training and usual care, and between resistance training and usual care, with Pilates training likely having the best efficacy. (Figs. 6 and Table 5, and in Additional file 1: Annexes 1.

**Table 5:**
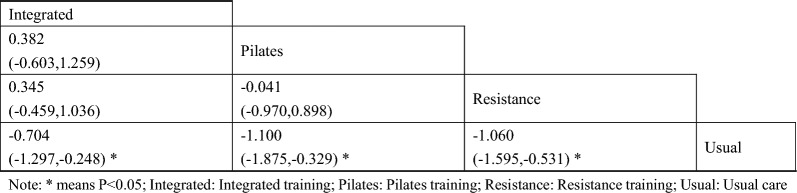
League table of different trainings on blood glucose two hours after meal in diabetic patients with frailty

#### Triglycerides

**Fig. 7:**
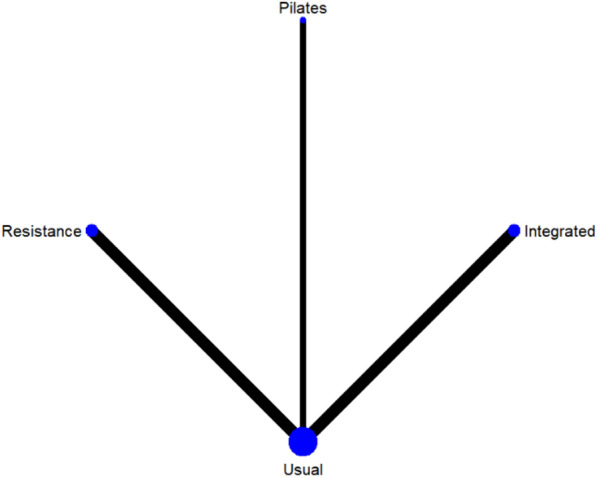
Network diagram of different training on triglycerides in diabetic patients with frailty

A total of 5 studies on triglycerides were included [43, 44, 47, 48, 50]. The results of network Meta-analysis showed that Pilates training [MD = − 0.86, 95%CI (− 1.37, − 0.33), P < 0.05] and resistance training [MD = − 0.85, 95%CI (− 1.20, − 0.50), P < 0.05] were better than the control group in reducing triglyceride. The triglyceride lowering effect of integrated training was lower than that of Pilates training [MD = 0.72, 95%CI (0.07, 1.31), P < 0.05] and resistance training [MD = 0.72, 95%CI (0.20, 1.18), P < 0.05]. The best probability ranking results showed that Pilates training (0.501) had the greatest effect on reducing triglyceride in patients with diabetic frailty compared with resistance training (0.494) and integrated training (0.004). this suggests that there is a statistically significant difference in the improvement of triglycerides between the Pilates training and usual care, and between the resistance training and usual care, with the Pilates training likely to have the best efficacy. (Figs. 7 and Table 6, Additional file 1: Annexes 1).

**Table 6:**
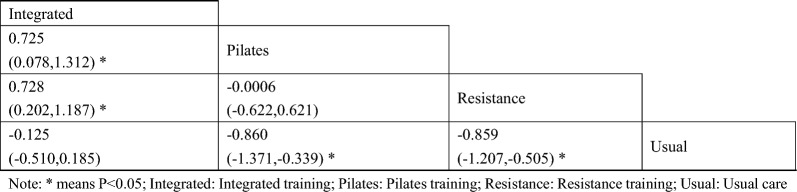
League table of different trainings on triglycerides in diabetic patients with frailty

#### Low-density cholesterol

**Fig. 8:**
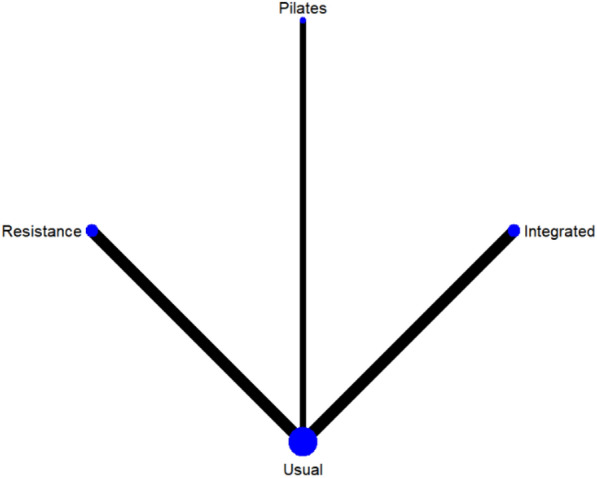
Network diagram of different training on low-density cholesterol in diabetic patients with frailty

A total of 5 studies on triglycerides were included [43, 44, 47, 48, 50]. The results of network Meta-analysis showed that there was no statistically significant difference in training therapy between any two groups (P > 0.05). The results of the best probability ranking showed that Pilates training had the best effect on improving low-density cholesterol in diabetic patients with frailty. this suggests that there is no statistically significant difference between various trainings and usual care in improving low-density cholesterol, while Pilates training may have the best efficacy. (Figs. 8 and Table 7, Additional file 1: Annexes 1).

**Table 7:**
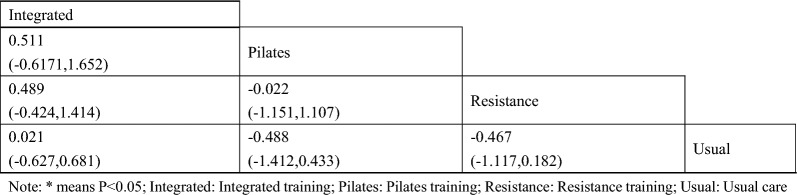
League table of different trainings on low-density cholesterol in diabetic patients with frailty

#### Short physical performance battery

**Fig. 9:**
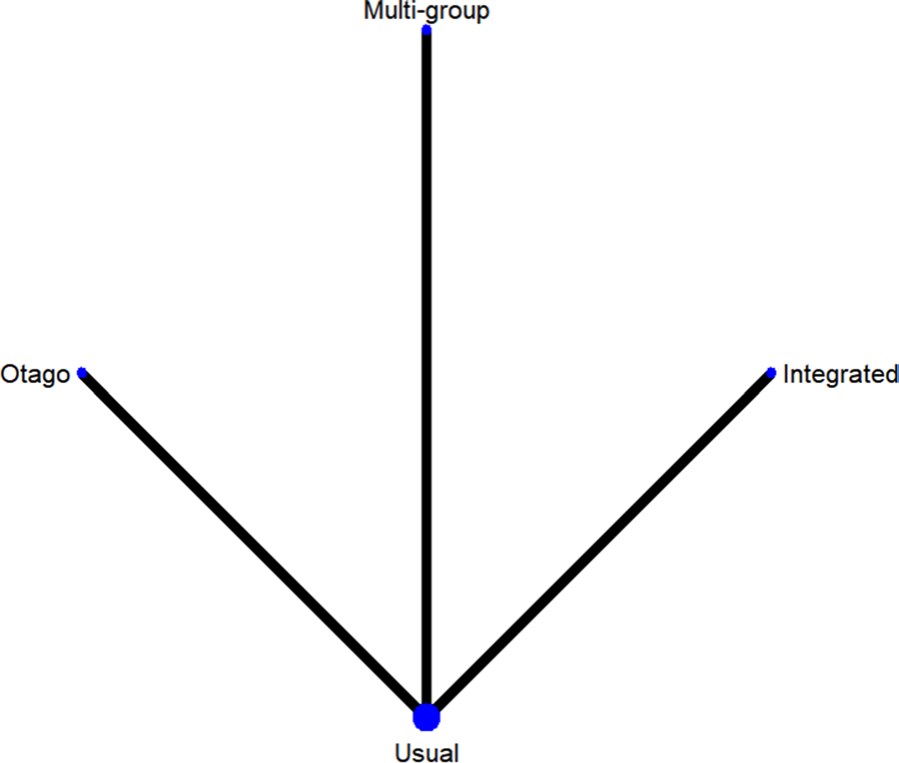
Network diagram of different training on SPPB in diabetic patients with frailty

A total of 3 studies on SPPB were included [38, 41, 43]. The results of network Meta-analysis showed that there was no statistically significant difference in training therapy between any two groups (P > 0.05). The results of the best probability ranking showed that multi-group training had the best effect on improving the SPPB score of diabetic patients with frailty. This suggests that there is no statistically significant difference between various trainings and usual care in improving SPPB, while multi-group training may have the best efficacy. (Figs. 9 and Table 8, Additional file 1: Annexes 1).

**Table 8:**
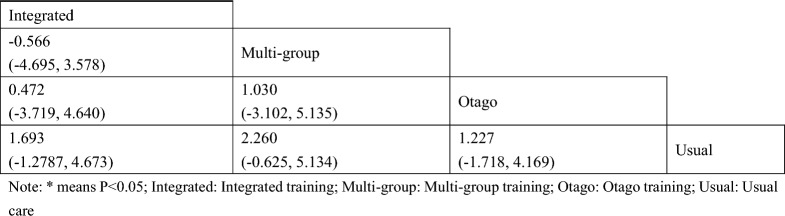
League table of different trainings on low-density cholesterol in diabetic patients with frailty

#### Body mass index

**Fig. 10:**
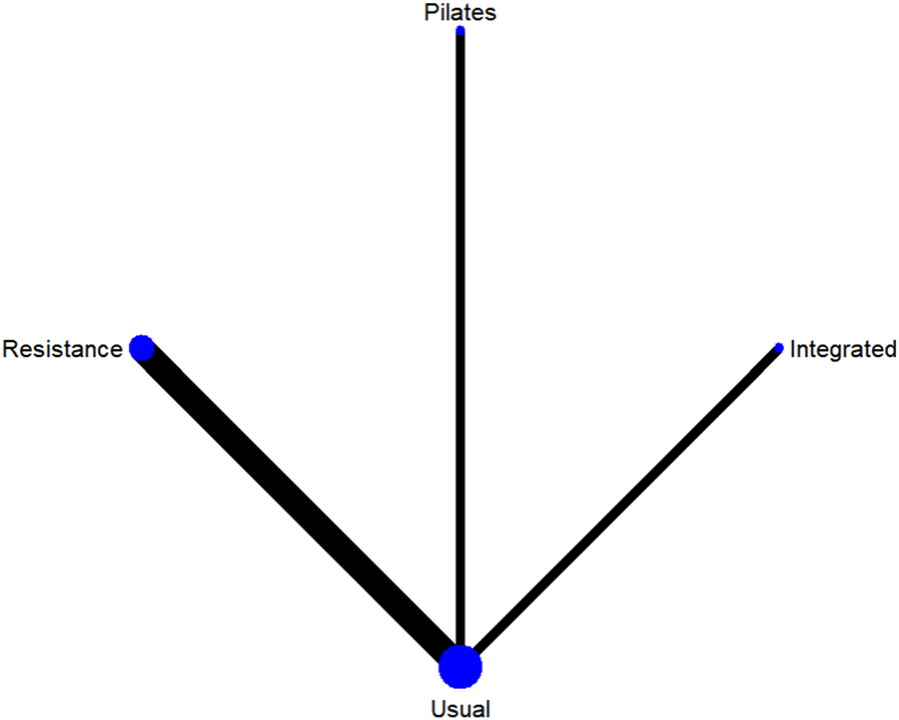
Network diagram of different training on BMI in diabetic patients with frailty

A total of 5 studies on BMI were included [44, 47, 48, 50, 51]. The results of network meta-analysis showed that the triglyceride lowering effect of resistance training [MD = − 2.19, 95%CI (− 3.66, − 0.62), P < 0.05] was better than that of the control group. There was no significant difference in training therapy between any two groups (P > 0.05). The results of the best probability ranking showed that Pilates had the best effect on improving BMI in diabetic patients with frailty. This suggests that there is no statistically significant difference between various trainings and usual care in improving BMI, while multi-group training may have the best efficacy. (Figs. 10 and Table 9, Additional file 1: Annexes 1).

**Table 9:**
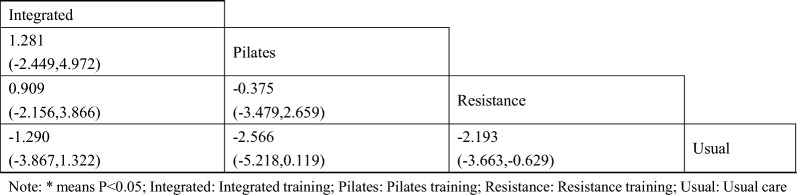
League table of different trainings on BMI in diabetic patients with frailty

### Inconsistency between direct and indirect comparisons

The literature included in this study has not yet formed a ring structure, so an indirect comparison method was used in this study. The consistency analysis was performed for each outcome index, and it was found that the scale reduction factor parameter value was close to 1.00, indicating good convergence. In data analysis, the *I*^2^ of all studies was less than 50%, the consistency model was used for network Meta-analysis.

### Publication bias

The improvement of fasting blood glucose and glycosylated hemoglobin in diabetic patients with frailty was represented by a funnel plot. The funnel plot shows that the points are not completely symmetrical, indicating that there may be some publication bias. (Additional file 1: Annexes 1).

## Discussion

This study included all current studies of exercise interventions in patients with diabetes mellitus and frailty in China through a network meta-analysis. By assessing the effects of various interventions, it is possible to determine which forms of exercise are the best way to improve the patient's health. The current evidence suggests that multi-group training is the best way to reduce fasting blood glucose and improve physical activity before meals, and Pilates training may be the best way to reduce glycated hemoglobin, blood glucose two hours after meals, improve blood lipid level and BMI in patients with diabetes in China. Interestingly, this study separated Pilates and Otago training from the other groups, which differed from the traditional way of differentiating (i.e., aerobic training, resistance training, and training combining aerobic and resistance training). In the results, we were surprised to find that Pilates training had a good effect on a variety of indicators in the study population, which seems to have been less mentioned in previous studies.

We hope that this result will be useful for the exercise treatment of patients with frailty in the clinic, or for exercise guidance in patients in the community. In the coming wave of ageing, movement can provide benefits not just confined to the patient’s health, it would also alleviate the pressure of the family to take care of, cultivate the habit of people movement, and the final result of the above will in medical related financial pressure and the citizen embodied overall health level. Therefore, we hope that the results of this study can contribute to the maintenance of patient’s health and the reduction of medical stress.

Diabetes is a global health problem, and people with diabetes are at high risk of frailty. People with diabetes face the challenge of exercising to control their blood glucose, and training is an important treatment for frailty. Therefore, it is particularly important to choose an training method suitable for diabetic patients with frailty to reduce blood glucose and improve physical function.

### The results of this study show that multi-group training has advantages in improving fasting blood glucose and physical activity ability of patients

Multi-group training is recommended by the American College of Sports Medicine as an effective alternative to traditional training training to improve the physical function of the elderly [53]. Studies [54, 55] have shown that multi-group training can not only regulate glucose metabolism and lipid metabolism in diabetic patients, reduce the risk of cardiovascular disease, but also improve frailty, cognitive function, emotional state of the elderly. However, there are few network meta-analyses including multi-group training interventions, and it is impossible to obtain the horizontal comparison results of multi-group training.

### The results of network Meta-analysis showed that Pilates training had certain advantages in reducing glycosylated hemoglobin, postprandial blood glucose, blood lipids and BMI in diabetic patients with frailty

Pilates training, invented by Joseph Hubertus Pilates, is a kind of training that integrates the training of body muscles and body functions with the concept of breathing and mental concentration. It is an training method that improves the body's strength, flexibility and core body control [56], while emphasizing the role of breathing on the body [57]. Previous studies have shown that Pilates training has a good effect on pain relief [58], while recent studies have shown that Pilates training has a good effect on improving the balance of the body, reducing the risk of falls [59], controlling blood pressure [60] and relieving bad emotions [61]. A study that included 21 subjects [62] showed that Pilates training was the best way to improve Proactive balance. However, another systematic review on elderly patients with type 2 diabetes [63] showed that Pilates training was superior to other trainings in improving glycosylated hemoglobin, cholesterol, triglyceride and other indicators. This study shows that integrated training is the best training for blood glucose control and lipid reduction. Other studies have shown that it is the best measure for lowering blood lipids and glycated protein [64, 65], but not Pilates training. This may be because: (1) There were not enough Pilates training in the included studies, but mainly divided into three categories: resistance training, aerobic training, and the combination of resistance training and aerobic training (integrated training); (2) Different patient population: previous studies mostly focused on patients with diabetes or type 2 diabetes, while this study focused on patients with diabetes and frailty.

### Other training methods included in this study have weak effects on blood glucose, blood lipids, physical training ability, BMI and other aspects

Although the American College of Sports Medicine recommends resistance training as a safe and effective lifestyle intervention for the elderly [66], and both resistance training and aerobic training are related to the improvement of systemic insulin sensitivity and blood glucose control in the elderly [67], their training action time is relatively short, and there is a certain gap compared with other training methods that take into consideration endurance training.

Otago training involves muscle strength, balance, walking and other aspects of training, which takes into account aerobic, resistance and balance, but it is a home-based, progressive training for the prevention of frailty and falls. It has been proved to effectively improve the cognitive function, balance ability, lower limb muscle strength and functional physical fitness of the elderly [68], and prevent the elderly from falling [69]. Accelerated recovery of physical function and reduced economic costs [70, 71] due to the greater emphasis on strength training and balance training. However, compared with other types of training, Otago training pays more attention to fall prevention [72, 73], so it may be inferior to other sports in terms of training volume and cannot provide enough stimulation to the body, so the effect is relatively weak.

Integrated training can take into consideration the requirements of strength training and endurance during training, which has been proved to be beneficial to the improvement of vascular function [74]. At the same time, it can improve cardiac dysfunction by reducing inflammation and oxidative stress [75]. Another study showed that integrated training is beneficial to the improvement of metabolic syndrome in patients. However, the lack of reasonable allocation of time arrangement in the implementation process of the included studies may affect the intervention effect. As for the research on frailty, some studies have concluded that resistance training has the greatest potential to improve frailty of patients [76]. However, all the studies included in this paper used resistance training for the intervention of frailty, so Meta-analysis is not possible.

## Limitations

Limitations of this study: (1)The inclusion of the literature in this study is limited to China, which may lead to the fact that the conclusions of this study cannot be broadly applied to other regions; (2)The number of articles included in this study was low and all comparisons were made only with “usual care”. Therefore, we were unable to make direct comparisons, which may have affected the validity of the results to some extent. In the future, we will include more direct comparative evidence of interventions; (3)There were some differences in the measurement and expression of the outcome indicators among the included studies, so the outcomes of such studies were not analyzed by Meta-analysis.

## Conclusion

Regarding the choice of the best exercise modality for Chinese patients with diabetes mellitus and frailty, this review presents the best evidence for the current lack of direct comparisons. The current evidence suggests that for diabetic patients with frailty in China, multi-group training may be the best training method for reducing fasting blood glucose before meals and improving physical performance, and Pilates training may be the best training method for reducing glycosylated hemoglobin, 2-h postprandial blood glucose, improving lipid levels and BMI. There are some limitations in the literature included in the study, and the above conclusions need to be verified by more studies.

### Supplementary Information

**Additional file 1:** Supplementary materials.

## Data Availability

The datasets used and/or analysed during the current study are available from the corresponding author on reasonable request.
