# Supplementary material for: Comparative effectiveness of different modes of exercise interventions in diabetics with frailty in China: a systematic review and a network meta-analysis
Source: Diabetol Metab Syndr. 2024 Feb 26;16:48. doi: 10.1186/s13098-023-01248-x (PMC10895831; doi:10.1186/s13098-023-01248-x)
Supplement: Supplementary file 1 — Additional file 1: Supplementary materials. [file 13098_2023_1248_MOESM1_ESM.zip › Additional file 1-Table 1 Results of literature search.docx]

| Table 1 Results of literature search | | |
| --- | --- | --- |
| Literature Library | Search queries | Search results |
| Pubmed | #1 Search: ((((frailty[MeSH Terms]) OR (frailty[Title/Abstract])) OR (frial elderly[MeSH Terms])) OR (frial elderly[Title/Abstract])) OR (frail*[Title/Abstract]) | 35520 |
|  | #2 Search: ((diabetes mellitus[MeSH Terms]) OR (diabetes mellitus[Title/Abstract])) OR (daibete*[Title/Abstract]) | 594,764 |
|  | #3 Search: (((((((((((((((((Exercise[MeSH Terms]) OR (Exercise[Title/Abstract])) OR (movement[MeSH Terms])) OR (movement[Title/Abstract])) OR (physical therapy[MeSH Terms])) OR (physical therapy[Title/Abstract])) OR (Exercise Therapy[MeSH Terms])) OR (Exercise Therapy[Title/Abstract])) OR (Exercise Movement Techniques[MeSH Terms])) OR (Exercise Movement Techniques[Title/Abstract])) OR (exercise*[Title/Abstract])) OR (movement*[Title/Abstract])) OR (aerobic*[Title/Abstract])) OR (resist*[Title/Abstract])) OR (resistant*[Title/Abstract])) OR (resistance*[Title/Abstract])) OR (combine*[Title/Abstract])) OR (combina*[Title/Abstract]) | 4,400,279 |
|  | #4 #1 and #2 and #3 | 257 |
| Web of sience | #1 (((((TI=(frailty)) OR AB=(frailty)) OR TI=(frial elderly)) OR AB=(frial elderly)) OR TI=(frail*)) OR AB=(frail*) | 47685 |
|  | #2 (((TI=(diabetes mellitus)) OR AB=(diabetes mellitus)) OR TI=(daibete*)) OR AB=(daibete*) | 330635 |
|  | #3 (((((((((((((((((((((((((TI=(Exercise )) OR AB=(Exercise )) OR TI=(movement )) OR AB=(movement )) OR TI=(physical therapy )) OR AB=(physical therapy )) OR TI=(Exercise Therapy)) OR AB=(Exercise Therapy)) OR TI=(Exercise Movement Techniques)) OR AB=(Exercise Movement Techniques)) OR TI=(exercise*)) OR AB=(exercise*)) OR TI=(movement*)) OR AB=(movement*)) OR TI=(aerobic*)) OR AB=(aerobic*)) OR TI=(resist*)) OR AB=(resist*)) OR TI=(resistant*)) OR AB=(resistant*)) OR TI=(resistance*)) OR AB=(resistance*)) OR TI=(combine*)) OR AB=(combine*)) OR TI=(combina*)) OR AB=(combina*) | 16705293 |
|  | #4 #1 AND #2 AND #3 | 198 |
| Embase | #1 frailty:ti,ab,kw OR 'frial elderly':ti,ab,kw OR frail*:ti,ab,kw | 52882 |
|  | #2 'diabetes mellitus':ti,ab,kw OR daibete*:ti,ab,kw | 376440 |
|  | #3 exercise:ti,ab,kw OR movement:ti,ab,kw OR 'physical therapy':ti,ab,kw OR 'exercise therapy':ti,ab,kw OR 'exercise movement techniques':ti,ab,kw OR exercise*:ti,ab,kw OR movement*:ti,ab,kw OR aerobic*:ti,ab,kw OR resist*:ti,ab,kw OR resistant*:ti,ab,kw OR resistance*:ti,ab,kw OR combine*:ti,ab,kw OR combina*:ti,ab,kw | 5005026 |
|  | #4 #1 AND #2 AND #3 | 192 |
| Cochrane library | #1 (frailty):ti,ab,kw OR (frial elderly):ti,ab,kw OR (frail*):ti,ab,kw | 5167 |
|  | #2 (diabetes mellitus):ti,ab,kw OR (daibete*):ti,ab,kw | 78218 |
|  | #3 (exercise*):ti,ab,kw OR (movement*):ti,ab,kw OR (aerobic*):ti,ab,kw OR (resist*):ti,ab,kw OR (combine*):ti,ab,kw | 367283 |
|  | #4 #1 AND #2 AND #3 | 80 |
| CNKI | ( ( ( ( ( ( 主题%='衰弱' or 题名%='衰弱' ) OR ( 关键词='衰弱' ) ) OR ( 摘要='衰弱' ) ) OR ( 题名='衰弱' ) ) AND ( ( ( ( 主题%='糖尿病' or 题名%='糖尿病' ) OR ( 关键词='糖尿病' ) ) OR ( 摘要='糖尿病' ) ) OR ( 题名='糖尿病' ) ) ) AND ( ( ( ( ( ( ( ( ( 主题%='运动' or 题名%='运动' ) OR ( 关键词='运动' ) ) OR ( 摘要='运动' ) ) OR ( 主题%='锻炼' or 题名%='锻炼' ) ) OR ( 关键词='锻炼' ) ) OR ( 摘要='锻炼' ) ) OR ( 主题%='训练' or 题名%='训练' ) ) OR ( 关键词='训练' ) ) OR ( 摘要='训练' ) ) ) | 132 |
| WANFANG | （主题:(衰弱) or 题名或关键词:(衰弱) or 摘要:(衰弱)） and （主题:(糖尿病) and 题名或关键词:(糖尿病) or 摘要:(糖尿病)） and （主题:(运动) or 题名或关键词:(运动) or 摘要:(运动) or 题名或关键词:(锻炼) or 摘要:(锻炼) or 题名或关键词:(训练) or 摘要:(训练)） | 84 |
| VIP | (M=衰弱 OR R=衰弱) AND (M=糖尿病 OR R=糖尿病) AND (M=运动 OR R=运动 OR M=锻炼 OR R=锻炼 OR M=训练 OR R=训练) | 69 |
| SinoMed | #1 "衰弱"[中文标题:智能] OR "衰弱"[摘要:智能] OR "衰弱"[关键词:智能] AND "衰弱"[加权:扩展] | 6396 |
|  | #2 "糖尿病"[中文标题:智能] OR "糖尿病"[摘要:智能] OR "糖尿病"[关键词:智能] OR "糖尿病"[加权:扩展] | 442800 |
|  | #3 "运动"[中文标题:智能] OR "运动"[摘要:智能] OR "运动"[关键词:智能] OR "锻炼"[中文标题:智能] OR "锻炼"[摘要:智能] OR "锻炼"[关键词:智能] OR "训练"[中文标题:智能] OR "训练"[关键词:智能] OR "训练"[关键词:智能] | 334478 |
|  | #4 #1 AND #2 AND #3 | 49 |
