# Supplementary material for: Comparative effectiveness of different modes of exercise interventions in diabetics with frailty in China: a systematic review and a network meta-analysis
Source: Diabetol Metab Syndr. 2024 Feb 26;16:48. doi: 10.1186/s13098-023-01248-x (PMC10895831; doi:10.1186/s13098-023-01248-x)
Supplement: Supplementary file 1 — Additional file 1: Supplementary materials. [file 13098_2023_1248_MOESM1_ESM.zip › Additional file 8-Figure 7 Glycated hemoglobin funnel diagram.docx]

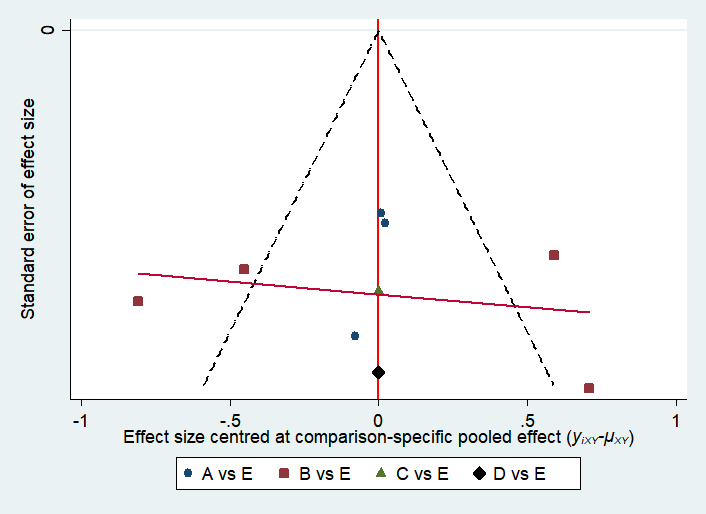


Figure 7: Glycated hemoglobin funnel diagram

Note：A=Resistance; B=Integrated; C=Multi-group; D=Pilates; E=Usual
