# Supplementary figures and images for: Comparative effectiveness of different modes of exercise interventions in diabetics with frailty in China: a systematic review and a network meta-analysis
Source: Diabetol Metab Syndr. 2024 Feb 26;16:48. doi: 10.1186/s13098-023-01248-x (PMC10895831; doi:10.1186/s13098-023-01248-x)

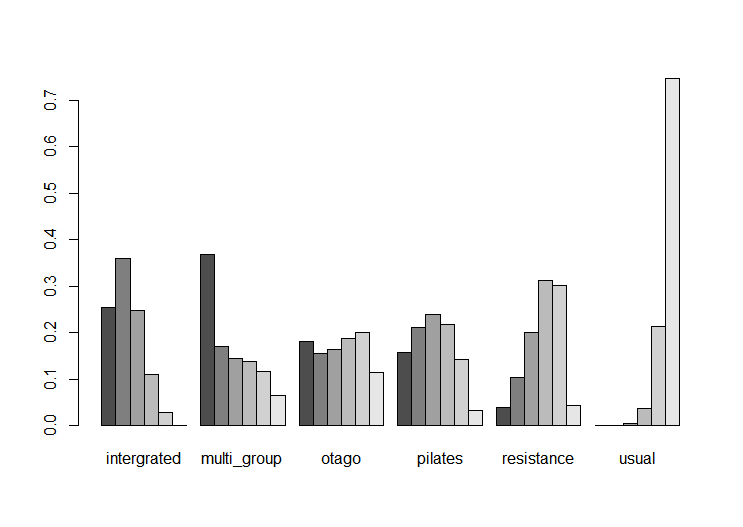


Figure 2:Fasting blood glucose ranking chart

Supplement: Supplementary file 1 — Additional file 1: Supplementary materials. [file 13098_2023_1248_MOESM1_ESM.zip › Additional file 3-Figure 2 Fasting blood glucose ranking chart.docx]

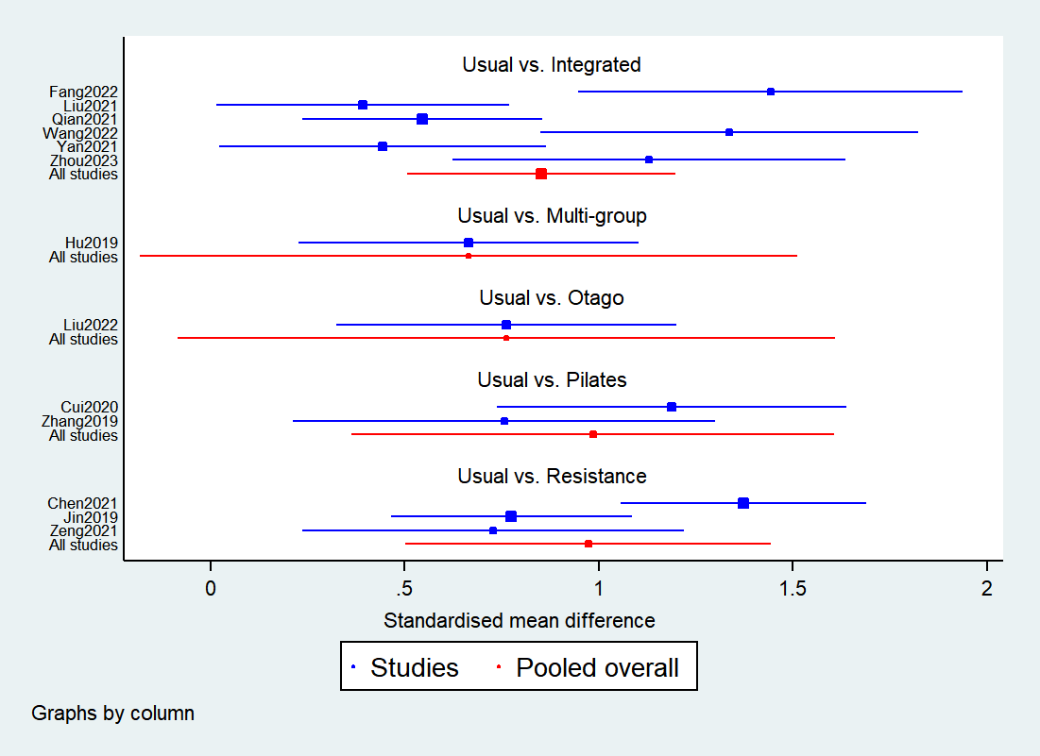


Figure 3: Fasting blood glucose forest plot

Supplement: Supplementary file 1 — Additional file 1: Supplementary materials. [file 13098_2023_1248_MOESM1_ESM.zip › Additional file 4-Figure 3 Fasting blood glucose forest plot.docx]

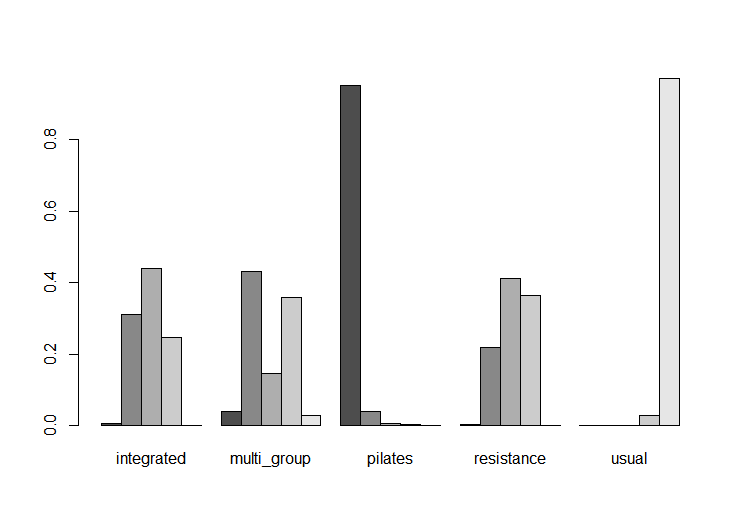


Figure 5: Glycated hemoglobin ranking scale

Supplement: Supplementary file 1 — Additional file 1: Supplementary materials. [file 13098_2023_1248_MOESM1_ESM.zip › Additional file 6-Figure 5 Glycated hemoglobin ranking scale.docx]

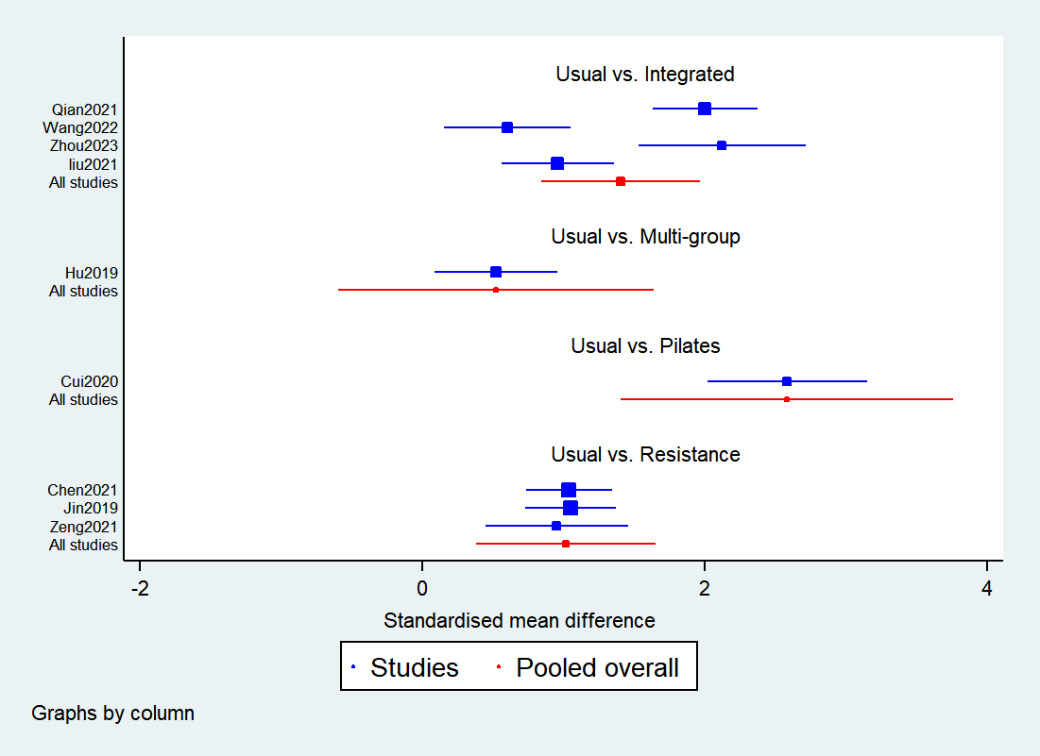


Figure 6: Glycated hemoglobin forest plot

Supplement: Supplementary file 1 — Additional file 1: Supplementary materials. [file 13098_2023_1248_MOESM1_ESM.zip › Additional file 7-Figure 6 Glycated hemoglobin forest plot.docx]

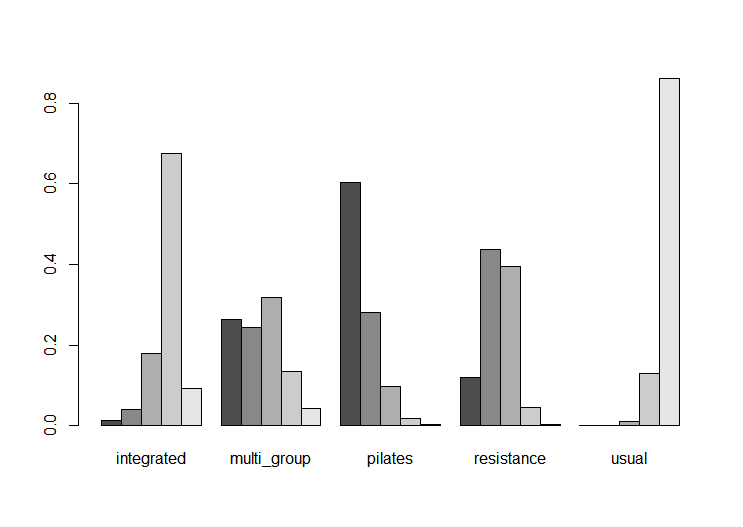


Figure 8: Postprandial glucose ranking scale

Supplement: Supplementary file 1 — Additional file 1: Supplementary materials. [file 13098_2023_1248_MOESM1_ESM.zip › Additional file 9-Figure 8 Postprandial glucose ranking scale.docx]

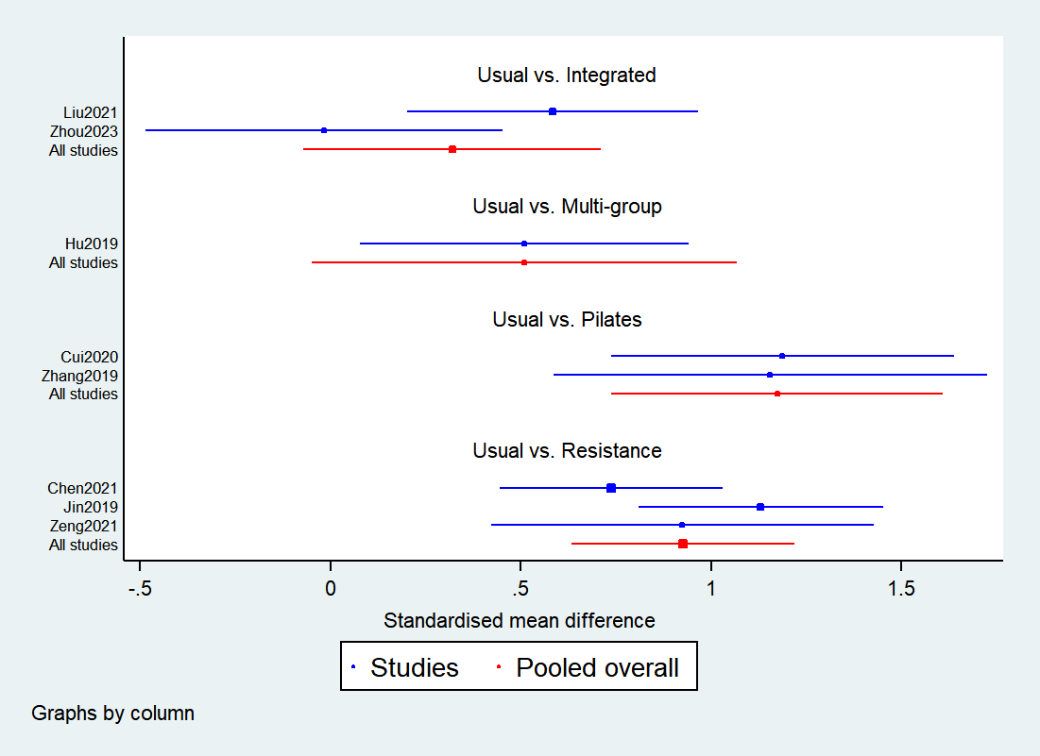


Figure 9: Postprandial blood glucose forest plot

Supplement: Supplementary file 1 — Additional file 1: Supplementary materials. [file 13098_2023_1248_MOESM1_ESM.zip › Additional file 10-Figure 9 Postprandial blood glucose forest plot.docx]

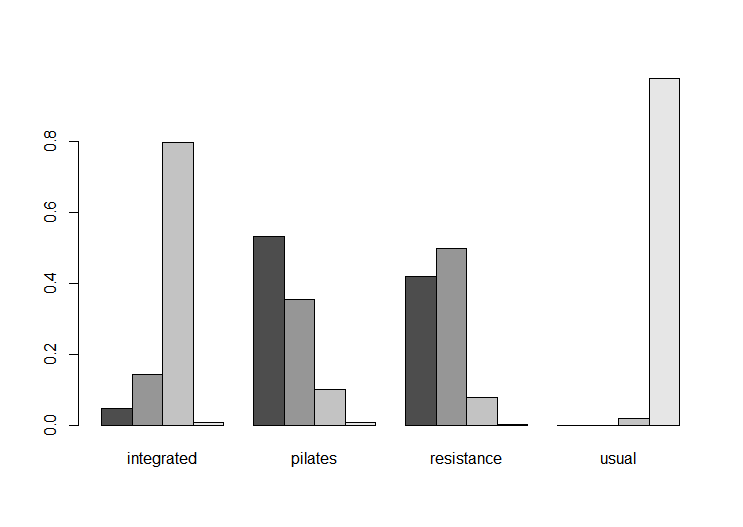


Figure 11: Total cholesterol sorting scale chart

Supplement: Supplementary file 1 — Additional file 1: Supplementary materials. [file 13098_2023_1248_MOESM1_ESM.zip › Additional file 12-Figure 11 Total cholesterol sorting scale chart.docx]

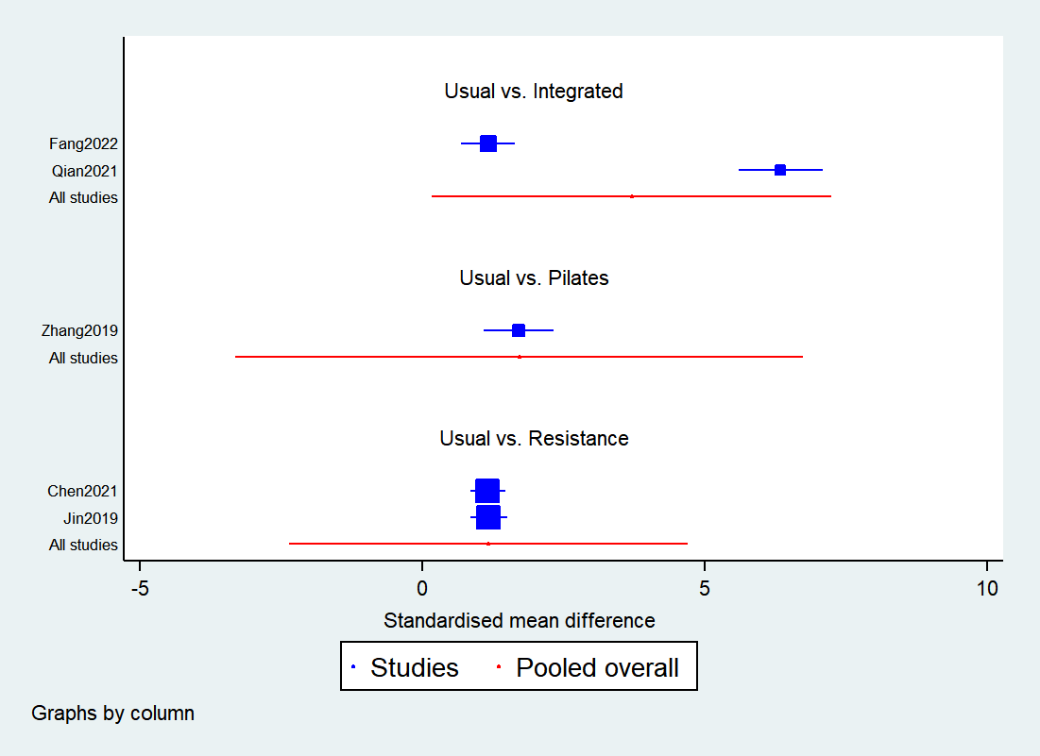


Figure 12: Total cholesterol forest plot

Supplement: Supplementary file 1 — Additional file 1: Supplementary materials. [file 13098_2023_1248_MOESM1_ESM.zip › Additional file 13-Figure 12 Total cholesterol forest plot.docx]

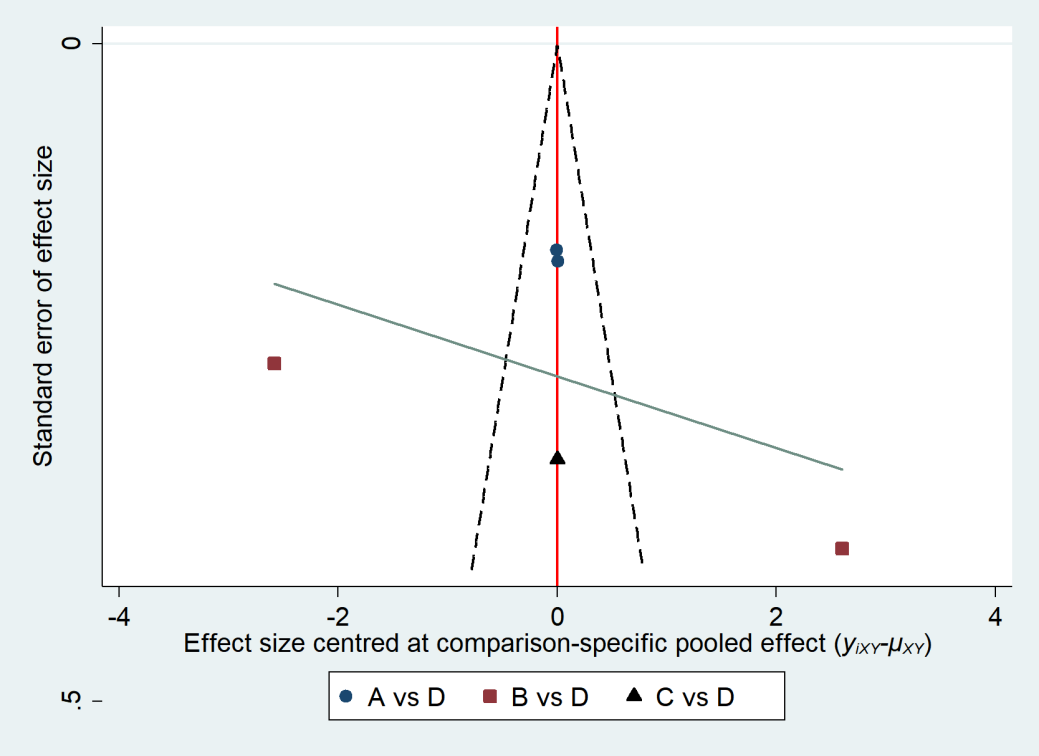


Figure 13: Total cholesterol funnel chart

Note：A=Resistance; B=Integrated; C=Pilates; D=Usual

Supplement: Supplementary file 1 — Additional file 1: Supplementary materials. [file 13098_2023_1248_MOESM1_ESM.zip › Additional file 14-Figure 13 Total cholesterol funnel chart.docx]

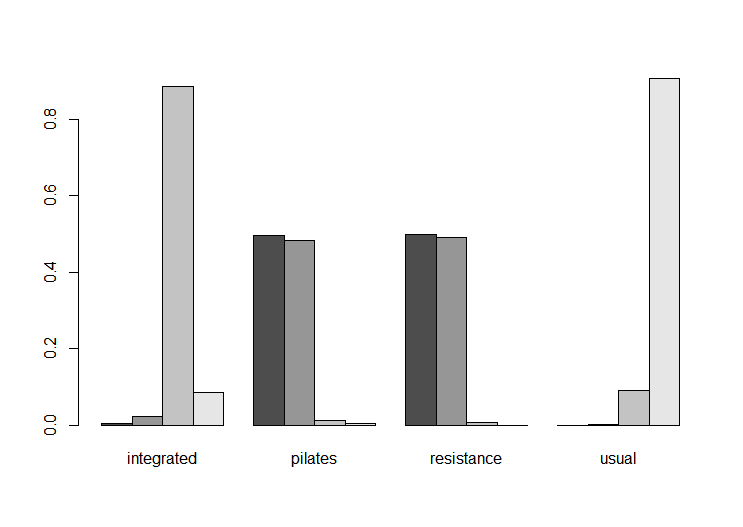


Figure 14: Triglyceride sorting grade chart

Supplement: Supplementary file 1 — Additional file 1: Supplementary materials. [file 13098_2023_1248_MOESM1_ESM.zip › Additional file 15-Figure 14 Triglyceride sorting grade chart.docx]

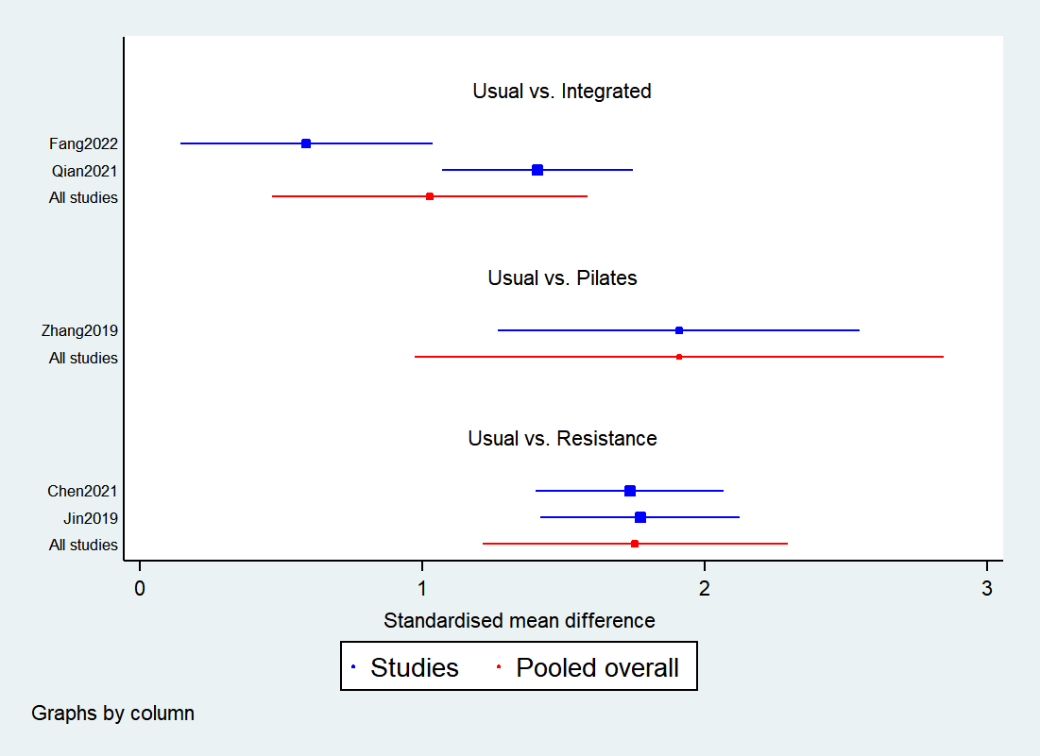


Figure 15: Triglyceride forest plot

Supplement: Supplementary file 1 — Additional file 1: Supplementary materials. [file 13098_2023_1248_MOESM1_ESM.zip › Additional file 16-Figure 15 Triglyceride forest plot.docx]

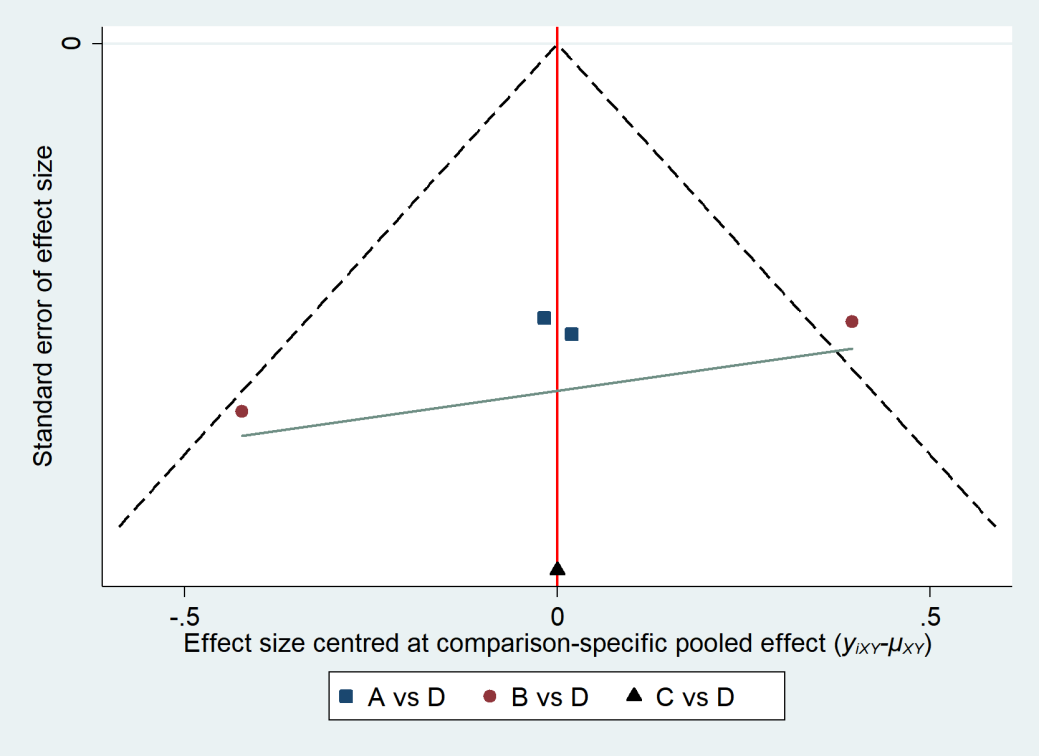


Figure 16: Triglyceride funnel diagram

Note：A=Resistance; B=Integrated; C=Pilates; D=Usual

Supplement: Supplementary file 1 — Additional file 1: Supplementary materials. [file 13098_2023_1248_MOESM1_ESM.zip › Additional file 17-Figure 16 Triglyceride funnel diagram.docx]

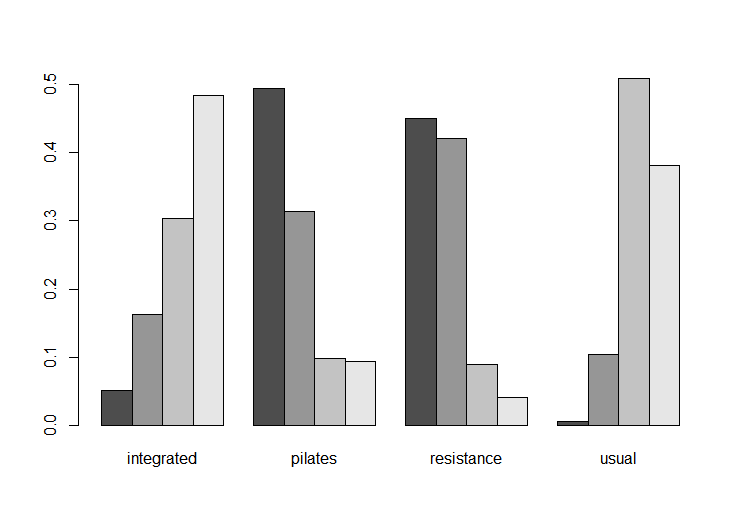


Figure 17: LDL sorting grade chart

Supplement: Supplementary file 1 — Additional file 1: Supplementary materials. [file 13098_2023_1248_MOESM1_ESM.zip › Additional file 18-Figure 17 LDL sorting grade chart.docx]

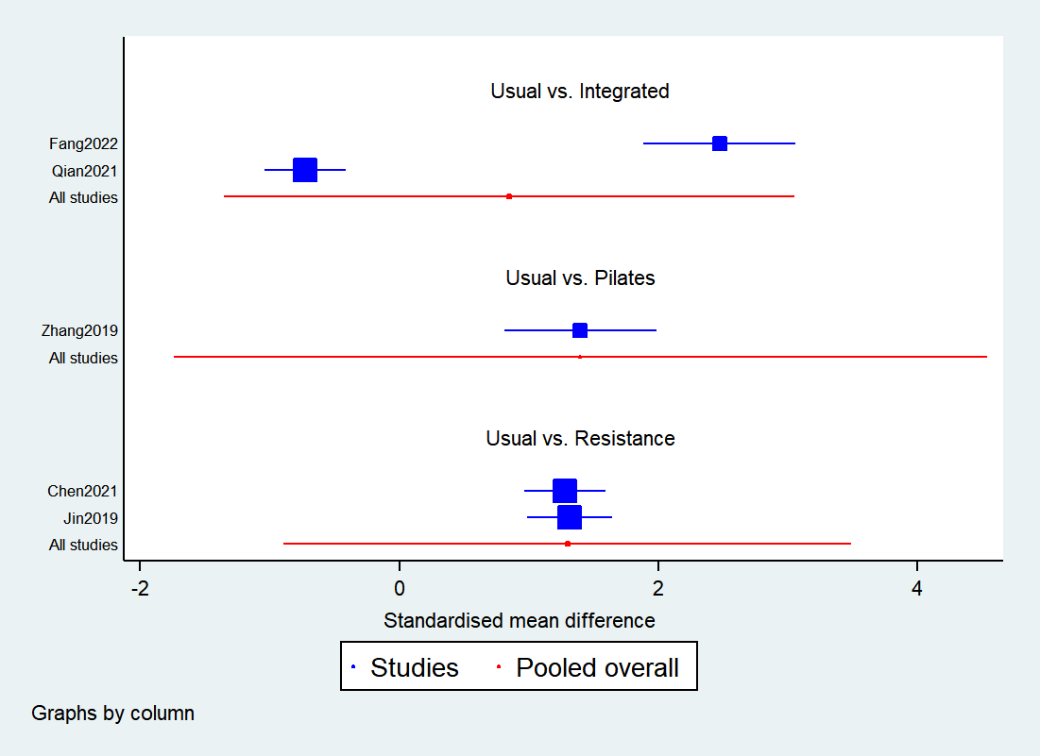


Figure 18: Low-density lipoprotein forest plot

Supplement: Supplementary file 1 — Additional file 1: Supplementary materials. [file 13098_2023_1248_MOESM1_ESM.zip › Additional file 19-Figure 18 Low-density lipoprotein forest plot.docx]

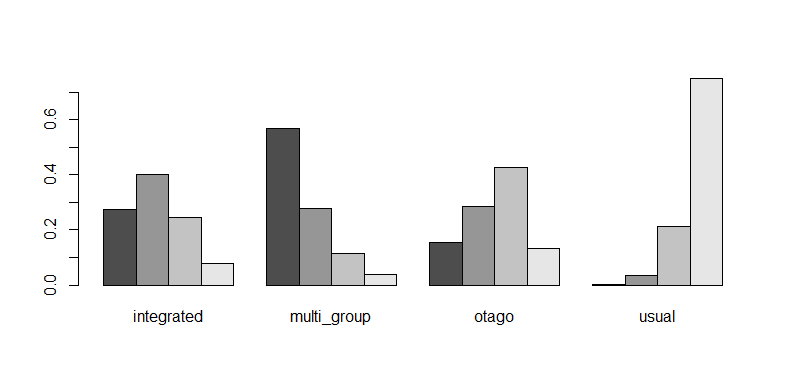


Figure 20: SPPB score sorting scale graph

Supplement: Supplementary file 1 — Additional file 1: Supplementary materials. [file 13098_2023_1248_MOESM1_ESM.zip › Additional file 21-Figure 20 SPPB score sorting scale graph.docx]

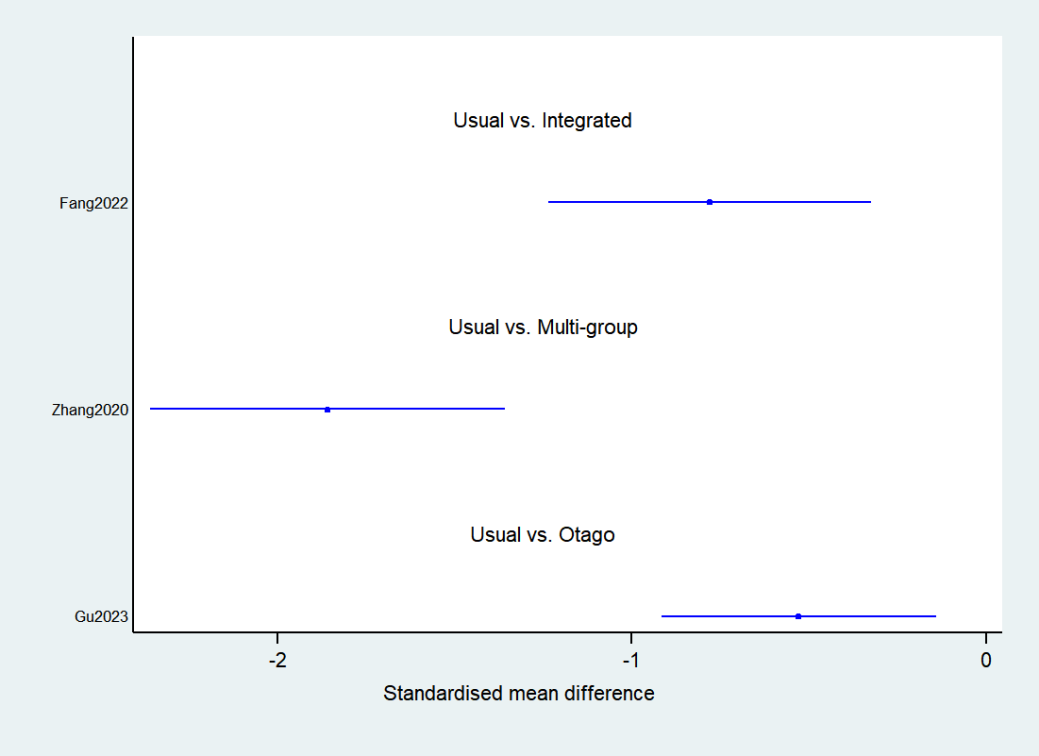


Figure 21: SPPB score sorting forest chart

Supplement: Supplementary file 1 — Additional file 1: Supplementary materials. [file 13098_2023_1248_MOESM1_ESM.zip › Additional file 22-Figure 21 SPPB score sorting forest chart.docx]

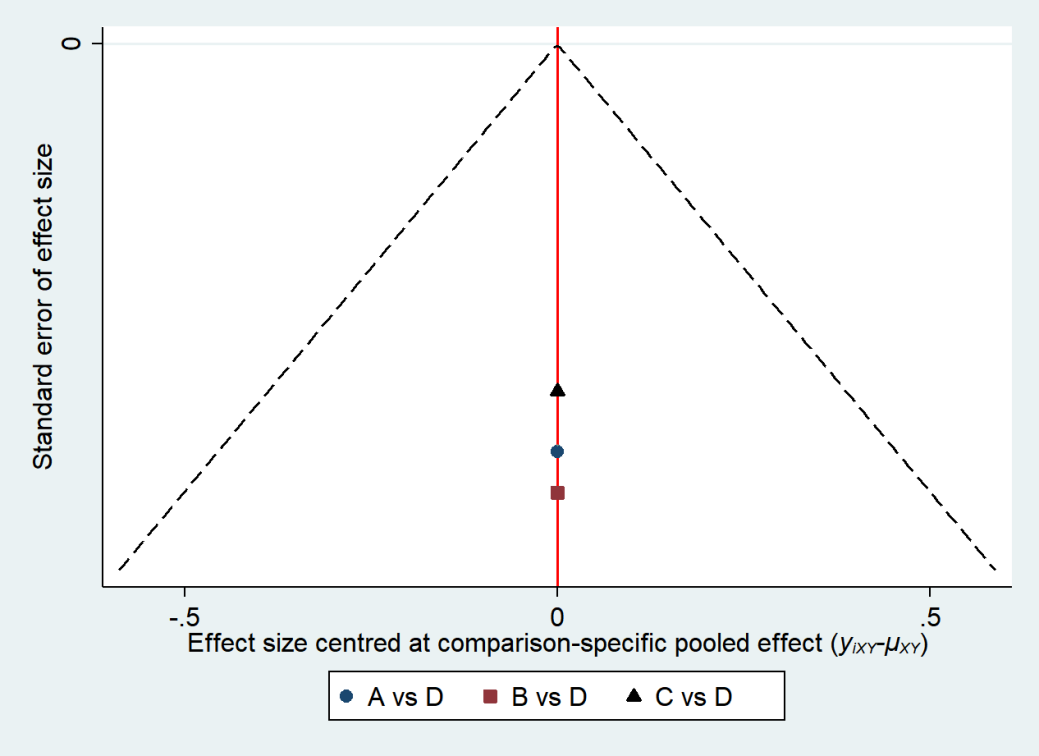


Figure 22: SPPB score funnel chart

Note：A=Integrated; B=Multi-group; C=Otago; D=Usual

Supplement: Supplementary file 1 — Additional file 1: Supplementary materials. [file 13098_2023_1248_MOESM1_ESM.zip › Additional file 23-Figure 22 SPPB score funnel chart.docx]

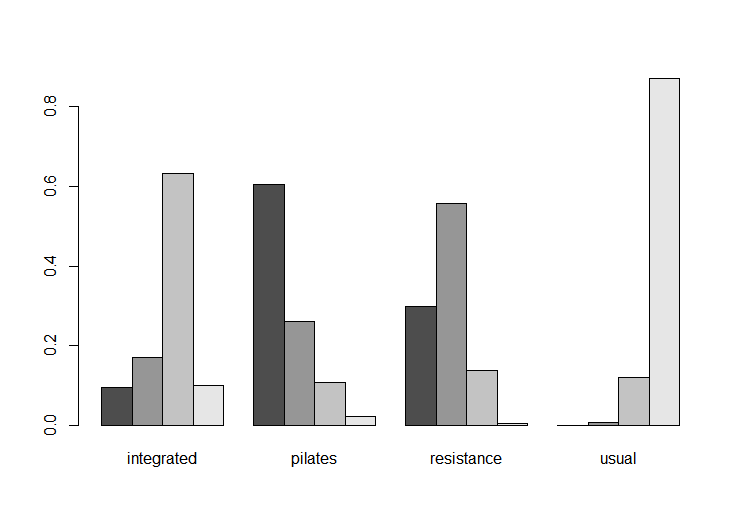


Figure 23: BMI score sorting graph

Supplement: Supplementary file 1 — Additional file 1: Supplementary materials. [file 13098_2023_1248_MOESM1_ESM.zip › Additional file 24-Figure 23 BMI score sorting graph.docx]

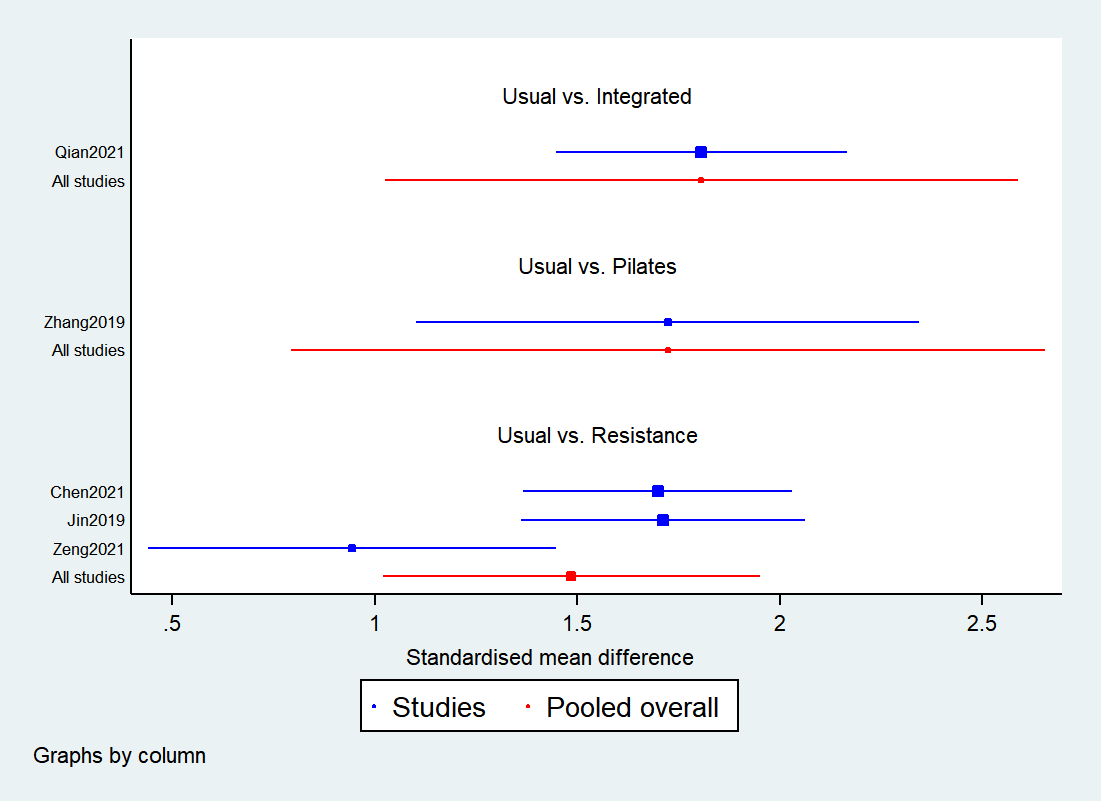


Figure 24:BMI forest map

Supplement: Supplementary file 1 — Additional file 1: Supplementary materials. [file 13098_2023_1248_MOESM1_ESM.zip › Additional file 25-Figure 24 BMI forest map.docx]

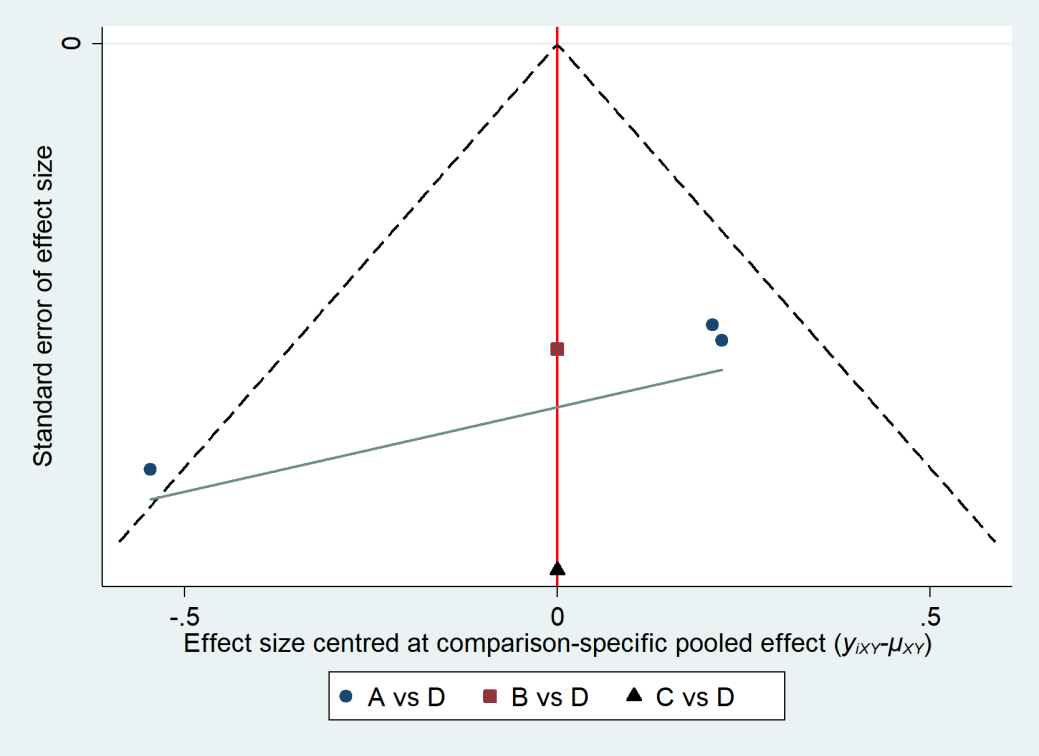


Figure 25: BMI funnel diagram

Note：A=Resistance; B=Integrated; C=Pilates; D=Usual

Supplement: Supplementary file 1 — Additional file 1: Supplementary materials. [file 13098_2023_1248_MOESM1_ESM.zip › Additional file 26-Figure 25 BMI funnel diagram.docx]
